# Body farms

**DOI:** 10.1007/s12024-017-9917-y

**Published:** 2017-09-15

**Authors:** Sue Black

**Affiliations:** Centre for Anatomy and Human Identification, Leverhulme Research Centre for Forensic Science, University of Dundee, Dundee, UK

The laudable intentions of science to research the effects of different variables on the ability to predict the time death interval accurately have become embroiled on multiple complex fronts. The colloquial and persistent use of the term ‘body farm’, the rhetoric that surrounds media reporting, and the sometimes distasteful display of the subjects, detract significantly from any scientific merit that the public may recognize from such a facility. Consequently, the voice of science may be struggling to compete with the noise of the media hype that surrounds such a facility. Perhaps we could try to simplify the landscape and change the nature of communication, if the voice of science is to be heard and respected.

Few forensic phrases have entered the public language more readily than ‘body farm’ which has captured imagination and been perpetuated in newspapers, magazines, online sites, films, novels and TV shows. Testing the temperature of the media’s portrayal can be achieved by a trawl of internet articles (the first port of call for the inquisitive public) and these seem to consistently start with words such as ‘gruesome’, ‘terrifying’ and ‘horrifying’ in the first sentence. Therefore, the first interaction that the public may have with this important research topic is wrapped in a frisson of sensationalism that will inevitably color that first impression. There is no doubt that decomposing rabbits and pigs are much less emotive than decomposing humans, and perhaps by permitting perpetuation of the rhetoric and exposure, we have secured a spectacular own goal. This is evidenced in an article arguing for the establishment of a future facility which has the opening line ‘imagine your dead grandmother lying in an open field, being attacked by vultures’ [1]. It is a deeply unpleasant concept which is regrettably reinforced by visuals as the public can opt for a ‘virtual tour’ of a facility online and there is no shortage of images and videos of human decomposition on all major internet platforms [2]. The overriding impression is one where the balance between serious informative education and public entertainment have become misaligned. Whether this can be explained through over exuberance on the part of researchers trying to engage with the public or maybe innocent naiveté of the nature of the press, what has emerged over the last 30 years is a complex landscape where, in the eyes of the public, emotive rhetoric may have eclipsed scientific justification. So can we redress, and if so, how?

If taphonomic facilities are to regain scientific credibility then they need to focus relentlessly and entirely on rigor, repeatability, accuracy, reliability and scientific experimentation that is underpinned by large sample sizes, multiple black box testing and robust statistical validity. That the US National academy [3] and the President’s Advisory Council [4] have publicly questioned the validity of almost all forensic science, means that we must adopt a more robust strategy if we are to influence and convince our funders and academic institutions that continued, or indeed new, investment in expensive taphonomic facilities is merited through a) providing gold star academic return, b) translating into robust evidence for the judiciary and c) being worth the reputational risk. This route will not be easy and nor will it be swift or cheap.

We need to be realistic regarding the likelihood of improving on the current state of the science. Human taphonomic facilities are expensive and for the research to reach accepted gold evidential standards, then the number of donor cadavers and the number of trial repeats in different environments and climates will be costly and we need to be ready for that and to be able to justify it. With a lack of research funding hampering most countries, we need to ask whether we are absolutely certain, given the variability that everyone accepts occurs when the human decomposes, that we can get substantially closer than we currently are, to a more accurate estimation of the time death interval? Although we know that pigs do not decompose at exactly the same rate as humans, are we so absolutely sure that the difference between humans and pigs justifies the choice and cost of one resource over the other?

We are often asked in the UK ‘why don’t you have a body farm’? Maybe the more appropriate question would be ‘why do we need a human taphonomic facility’? Where is the incontrovertible large-scale evidence that animal facilities are not good enough? We have had 35 years of research from human taphonomic facilities, if we still don’t have the answer to this core question after that length of time, maybe we are asking the wrong question or perhaps we are simply have unrealistic expectations.
